# European Correspondence

**Published:** 1877-10

**Authors:** N. J. Nunn

**Affiliations:** London


### EUROPEAN CORRESPONDENCE.

London, October 2d, 1877.

*Messrs. Editors Atlanta Medical and Surgical Journal:*

It has long been the custom among medical schools, to open the season of teaching with what they have been pleased to term an introductory lecture. Sometimes this lecture takes the shape of a biography, serving no practical purpose but to show the research of the speaker, and test the patience of the audience. Sometimes the lecturer delivers a sermon on the duties of physicians, good enough, no doubt, for those who, having completed their education, are about to enter on the active work of their profession, but which can make no possible impression upon the young men who have assembled to store their minds with the facts necessary for an examination, of which this subject forms no part. On them, and many kindred subjects, much valuable time is wasted, while the number of introductory lectures devoted to the discussion of scientific subjects, is exceptionally small. One of these bright exceptions was the introductory delivered at King's College, by Prof. Lister, who, having accepted a chair in this institution, has moved to London from Edinburgh.

The first of October was the great "opening day"—to borrow a trade phrase—of the London medical schools, and although all, or nearly all, the introductories took place at the same hour, still the lecture theatre of King's College was densely crowded long before the appointed hour, by an audience drawn hither by the world-wide reputation of the lecturer

And here it may not be out of place to mention, that the younger portion of the assembly was much more boisterous and ill-behaved than is usually the case in American schools; so much so, that the speaker had, more than once, to administer a well-merited rebuke.

It was the generally received opinion, that the subject of the lecture would have some connection with the principles governing the application of antiseptics in surgery, with which the name and world-wide reputation of the lecturer are so intimately connected; but in place of that, the Professor chose for his theme some original investigations on the subject of fermentation, the result of which was, to prove, almost conclusively, that the fermentation, souring or development of lactic acid in milk is dependent on a special ferment, to which he has given the name of "bacterium lactis."

Almost any one of the old school doctors, if sitting as an examiner, would reject, as an arrant ignoramus, the man who didn't know that blood coagulated upon exposure to the air. Why, everybody knows that. It was only necessary to put fresh blood in a basin, and the change took place before your eyes. Quite true, it has that appearance; but this is only one more added to the millions of hasty conclusions, errors of diagnosis, which are the result of ignorance and incomplete investigations, which again, are not unfrequently the natural outcome of laziness, inattention, or criminal negligence. There appears to be nothing fixed in science. Things which formerly were accepted as almost axiomatic, are now proven to have been only deceptive appearances. The truths of to-day will be the errors of to-morrow; and so must the living tree of human knowledge increase, feeding on the mistakes of the dead past. Hereafter, the answer of every scientific man to a scientific opinion should be, "So far as investigations have come to my knowledge, such, appears to me, to be the case—mark you, only 'appears to me.'" Henceforth, let there be no intolerant positiveness in science. But to return to the lecture.

Among the first specimens shown by Prof. Lister, was a flask, about half full of blood, which was as fluid as when first introduced into the flask several weeks before. In this experiment, the flask having in the neck a pledget of cotton, through which a tube was passed into the flask, a few inches being left free outside, was carefully purified, and then the Prefessor, not having before his eyes a wholesome dread of "the Society for the Prevention of Cruelty to Animals," opened the jugular vein of an ox, and passing into it the tube connected with the flask, withdrew about a pint of blood. This was the specimen shown, and the air had to it free access through the pledger of cotton in the neck. Yet there it was, apparently in exactly the same condition as when first drawn—no coagulum, no change of color, (save a faint reddening on the surface, presumably from the action of the oxygen of the air), and no unpleasant odor. To one not abreast with the most recent investigations, this would appear little short of a miracle. This specimen was similar to one shown before the British Medical Association, at Manchester, by Dr. Roberts, of whose address you have already had a resume.

More prone than blood even to change, is a mixture of blood and water; yet here was another flask, containing some water, which, having been first boiled and cooled with proper precaution, had received an addition of some of the blood from the flask first shown. This flask, like the other, was simply stopped with cotton, so that the air had free access to the contents; yet here, as before, the fluid had apparently undergone no change.

A flask half filled with boiled milk was next shown, the stopper, as before, being cotton wool; but, although the specimen was several weeks old, the milk gave no evidence of change. This recalls experiments made by myself many years ago, on the preservation of milk, of course keeping in view the then orthodox opinion that the air was the cause of the changes which take place in that fluid, when exposed under ordinary conditions. A quantity of milk was placed in a bottle, enough oil poured on top to nearly fill the bottle, and the whole heated until the milk boiled. A bit of cotton was then put in, to exclude the dust, and the whole put aside to cool. It is needless to say the experiment was successful, but was looked upon according to the theories then in vogue, as merely the result of the exclusion of oxygen. How often are we unconsciously on the very threshold of truth, but too ignorant to take advantage of our position.

This milk specimen was the last of the series closed with cotton stoppers. The next set was most extraordinary, in that each glass containing a specimen was simply closed with a loose glass cap, and one or more of these glasses, sometimes to the number of twelve, were placed on a piece of plate glass and covered with a bell glass, which was freely and frequently removed and replaced.

It will appear that Professor Lister is not to be numbered among the supporters of the germ theory—at least to a very limited extent only. That is to say, he believes fermentations to be the result of the presence of some *variety of bacteria;* but he is not prepared to admit the existence of a bacterium germ in the absence of more positive proof, and in view of the fact that the known manner of the increase of bacteria is quite sufficient, he thinks, to satisfactorily account for all the phenomena which accompany their presence.

The effort of Dr. Roberts at Manchester, of which you have had an abstract, was an attempt to show that certain fevers were accompanied by the presence of certain microscopic organisms in the blood; and now Mr. Lister, traveling another of the converging roads, some of which he has already passed over, comes forward to prove:

1st. That the fermentation of milk is always accompanied with the presence of a special bacterium.

2d. That the presence of this special bacterium is always accompanied by fermentation in milk.

3d. That other bacteria, although appearing in certain decompositions of milk, are not accompanied by the lactic fermentation.

A microscopic specimen of the boiled milk just shown, demonstrated the entire absence of bacteria, and at the same time the want of any fermentive action in it. This was one step in advance; but to this experiment it might be objected that the boiling destroyed the preventive principle. Mr. Lister, therefore, determined to make a series of investigations on raw milk.

A quantity of milk was drawn from a cow into a prepared vessel, and thence transferred to a dozen test tubes, each of which was fitted with a loose tube cover, and a bell glass placed over the whole. In a few days they all soured. This was discouraging; but it must be mentioned that the experiment was partly performed in a cow house, the atmosphere of which was, doubtless, laden with bacteria. So it was determined to make another effort—this time in the open air—but the atmospheric conditions were unfavorable, and failure was the result this time also. A third trial was made, this time with increased precautions, and the result was most successful, the milk remaining perfectly sweet in some of the glasses. In some of the specimens other fungi appeared, but there was no souring, unless the bacterium lactis (Lister) was present.

The next step in the investigation was to ascertain the results of the inoculation of known quantities of milk with known numbers of bacteria, and for this purpose it was necessary to estimate the number of bacteria in a given quantity of milk. By means of a delicate syringe, graduated to one-hundredth ($\frac{1}{100}$) of a drop, it was ascertained that a microscopic cover, half ($\frac{1}{2}$) an inch in diameter, was exactly filled by one-fiftieth ($\frac{1}{50}$) of a drop. The number of fields of the object glass in the cover being known, it was only necessary to count the bacteria in one field, multiply by the number of fields in the cover, and the result would be the number of bacteria in one-fiftieth ($\frac{1}{50}$) of a drop. A quantity of milk was then diluted so as to contain one bacterium in each drop. Specimens of milk known to be free from bacteria were then inoculated with one, two and four drops of the solution, and the results were such as might be anticipated—the number of specimens souring bearing a proportion to the number of drops of solution employed; for while some in which one drop was used escaped, all of those in which four (4) drops were put, soured. All of those that soured showed the *bacterium lactis*, (Lister) while those that did not sour, showed none.

Now, it is absolutely demonstrated by these experiments, that the fermentive principle of milk is not a solution, for then every inoculated specimen would have fermented; but the Professor was prepared to admit, for the sake of argument, that the ferment, though non-living, was endowed with the power of self-multiplication as rapidly as the living bacterium; in other words, that the chemical lactic ferment, and the bacterium lactis, were mere accidental concomitants. Then, it would be necessary to imagine the two substances accidentally present should be present in precisely the same number, and that they would always be found in pairs—a bacterium lactis and a particle of chemical ferment. Both these positions the speaker looked upon as inconceivable, and he likewise regarded the exact proportion which he found to exist between the number of glasses fermenting and the number of bacteria counted, as indicating the absence of spores or germs for this species of bacterium.

In the course of the lecture, it was shown that the bacterium lactis refuses to live in Pasteur's solution, and this was regarded as conclusive proof that the bacterium lactis is a different variety from the other bacteria, which thrives in this fluid.

The bacterium lactis the lecturer defined a motionless bacterium—a microscopic fungus, multiplied by fissiparous generation, always by lines transverse to the longitudinal axis of the organism.

In the specimens which did not undergo lactic fermentation, a variety of organisms were found—bacteria and torulæ. One of these organisms, which appeared as little orange specks, was examined and was found to consist of granules differing from bacteria in this respect: that the only evidence of vitality was by seeing them under crucial fissiperous development. These bodies he has named granuligera.

Such is the gist of the lecture of Professor Lister, as understood by me; but some of the views entertained, some of the results of the experiments and some of the deductions and conclusions arrived at, appear so much at variance with the views of other investigators, that I think it best to reserve any comments I might wish to make, until I have seen a printed copy of the address. N. J. Nunn, M.D., &c.

